# ^18^F-DCFPyL PET/CT in Newly Diagnosed Prostate Cancer: Diagnostic Value of Intraprostatic PSMA Uptake in Risk Classification of Prostate Cancer

**DOI:** 10.3389/fonc.2022.800904

**Published:** 2022-02-03

**Authors:** Shuoming Zhou, Tiantian Liu, Ziqiang Zhu, Lin Zhang, Subo Qian, Hongliang Fu, Qifeng Cao, Jian Kang

**Affiliations:** ^1^Department of Urology, Xinhua Hospital Affiliated to Shanghai Jiao Tong University School of Medicine, Shanghai, China; ^2^Department of Urology, Anhui Provincial Children’s Hospital/Children’s Hospital of Fudan University (Affiliated Anhui Branch), Hefei, China; ^3^Department of Nuclear Medicine, Xinhua Hospital Affiliated to Shanghai Jiao Tong University School of Medicine, Shanghai, China

**Keywords:** prostate cancer, PSMA PET/CT, intraprostatic PSMA uptake, risk classification, quantitative parameters

## Abstract

**Purpose:**

^18^F-DCFPyL prostate-specific membrane antigen (PSMA) PET/CT is commonly applied to locate lesions of prostate cancer (PCa), but its diagnostic function of quantitative parameters is ignored. Our study evaluates the parameters of intraprostatic PSMA uptake in patients newly diagnosed with PCa and explores their predictive value in risk classification, which is similar to D’Amico criteria.

**Materials and Methods:**

We quantified the maximal standardized uptake value (SUVmax), mean SUV (SUVmean), total lesion (TL)-PSMA, prostate/muscle (P/M) ratio of the primary tumor, and PSMA-derived tumor volume (PSMA-TV) from 62 patients with histologically proven PCa. Patients newly diagnosed with PCa were allocated into risk groups (at low, intermediate, and high risk, respectively) in accordance with D’Amico criteria. Afterwards, the five parameters mentioned above among three different risk groups were compared, and their predictive values in the risk classification of PCa were explored.

**Results:**

Significantly decreased levels of SUVmax, SUVmean, TL-PSMA, and P/M ratio were observed in the risk groups of low or intermediate or both, compared with the high-risk group. However, only the P/M ratio significantly elevated in patients with intermediate risk [mean ± SD (median): 46.58 ± 9.74 (45.27), *P* = 0.042] or high risk [98.95 ± 38.83 (97.52), *P* < 0.001], compared with low-risk patients [12.33 ± 5.93 (9.81)]. When P/M ratio was used to distinguish between low-risk and intermediate-risk patients, its *c*-statistics was 0.660. On the other hand, when distinguishing between intermediate-risk and high-risk groups, the *c*-statistics of P/M ratio was 0.667. Finally, when P/M ratio was used to distinguish between low-risk and high-risk patients, the *c*-statistics was 0.969. P/M ratio had a positive correlation with prostate-specific antigen in all enrolled PCa patients.

**Conclusion:**

The quantitative parameters of ^18^F-DCFPyL PET/CT, including SUVmax, SUVmean, and P/M ratio, might assist in distinguishing low-risk or intermediate-risk groups from the high-risk group. Of these parameters, P/M ratio appears to be the better promising parameter for risk classification of prostate cancer than SUVmax.

## Introduction

Prostate cancer (PCa) is a common male malignancy worldwide and is the second leading cause in men who die of cancer in the Western world (1). It is reported that men have a 14% possibility of developing prostate cancer in their lifetime (2). Therefore, it is important for newly diagnosed patients to diagnose prostate cancer correctly and rank its severity. A previous meta-analysis has pointed out that conventional imaging techniques like CT and magnetic resonance imaging (MRI) have a sensitivity of 39%–42% and a specificity of 82% (3) when used in the diagnosis of prostate cancer. In this regard, prostate-specific membrane antigen (PSMA) PET/CT becomes an advantageous imaging method to detect prostate cancer lesions, which has a sensitivity of 67% to 97% (4). It even changed 50% to 87% of clinical treatment plans, which were previously based on the results of choline PET/CT examination (5, 6).

PSMA is a transmembrane folate hydrolase composed of 750 amino acids (7). The glycosylated transmembrane protein has higher expression in the majority of malignant prostate cells than that in the non-malignant prostate tissue. In addition, the expression of PSMA in PCa is related to increased prostate-specific antigen (PSA) levels and Gleason score (8). We know that, according to D’Amico criteria (9), we can stratify the newly diagnosed prostate cancer patients by Gleason sum, prostate-specific antigen, etc. into low-, intermediate-, or high-risk groups to guide follow-up treatment. At the same time, we want to figure out whether the semiquantitative PET values from ^18^F-DCFPyL PET/CT can be used for risk stratification in newly diagnosed PCa patients like Gleason scores and PSA levels. As far as we know, there are few related studies. So, we compared the maximal standardized uptake value (SUVmax), mean SUV (SUVmean), total lesion (TL)-PSMA, PSMA-derived tumor volume (PSMA-TV), and prostate/muscle (P/M) ratio of primary PCa patients with different risks to find the more potential parameter (10–12).

Therefore, this study aimed to appraise the diagnostic value of intraprostatic PSMA uptake in risk classification of exclusively untreated, newly diagnosed PCa patients receiving ^18^F-DCFPyL PET/CT that were confirmed by the later MRI/transrectal ultrasound (TRUS)-fusion biopsy or the specimens after prostatectomy. In the end, we further explored the correlation between SUV measurements and clinical parameters.

## Patients and Methods

### Enrollment of Participants

From September 2017 to March 2020, 256 unselected PCa patients who underwent ^18^F-DCFPyL PET/CT at the Department of Nuclear Medicine, Xinhua Hospital Affiliated to Shanghai Jiao Tong University School of Medicine were preliminary enrolled in the research. ^18^F-DCFPyL PET/CT was used to primarily stage newly diagnosed PCa patients, localize biochemical relapse after curative treatment, or systemically evaluate castration-resistant PCa. Patients were further screened in accordance with the following criteria: a) biopsy-proven, exclusively untreated, and newly diagnosed prostate cancer patients; b) with complete clinical data (such as BMI, PSA, CT, MRI, and bone scintigraphy); and c) the medical condition and vital signs of the patients were stable to lie supine for imaging. Finally, we enrolled 62 patients who met the above criteria for this study. Based on D’Amico criteria, patients newly diagnosed with PCa were allocated into different risk groups including low, intermediate, and high risks. All enrolled patients were notified of the investigation procedures and objectives, and informed consent was obtained (12).

### PSMA PET/CT Scanning

The synthesis of the PSMA PET tracers was performed as for ^18^F-DCFPyL in previous research (13). After 4 h of fasting, ^18^F-DCFPyL PET/CT was implemented. The injection of ^18^F-DCFPyL was scaled by body mass index (BMI), which ranges from 233 to 374 MBq. After an uptake period of 120 min, patients were scanned on the Biograph 16 TruePoint PET/CT scanner (Siemens, Germany) from the top of the skull to the middle thigh. The non-contrast-enhanced (low-dose) CT scan was implemented for positioning and attenuation correction (120 kV, automatic mA selection of 25–200 mA, and a pitch of 0.95). Immediately after CT scanning, PET data were obtained using 3-min acquisition time per bed position.

Afterwards, the ordered-subset expectation-maximization algorithm (OSEM) was applied to reconstruct CT-derived attenuation-corrected images. Finally, the attenuation-corrected ^18^F-DCFPyL PET/CT fused images were reconstructed in the horizontal plane, coronal plane, and sagittal plane, respectively (12).

### Qualitative and Quantitative Image Interpretation

The ^18^F-DCFPyL PET/CT images were obtained at 120 min after injection, and at least one nuclear medicine specialist and one radiologist scored PCa depositions and their accompanying anatomic locations using visual analysis. Primary PCa is diagnosed by an accumulation of focal tracer found in the prostate fossa, lymph nodes, or the distant site higher than that in the soft tissue surrounding the prostate, perirectal adipose tissue, or pelvic muscle, excluding physiologic absorption of the prostate (12). Physicians provided the qualitative interpretation case reports to record the number of positive lesions (0, 1, 2, 3, 4, 5, or >5) and the location of lesion (prostate, lymph nodes, skeletal manifestations, liver, or other).

Subsequently, the extraction of quantitative data was carried out by two experienced nuclear medicine physicians at a post-processing workstation. All scans were evaluated visually. Pathological uptakes were initially assumed if a lesion showed a tracer uptake higher than the local background (14). Depending on the localization, they were rated as local (prostate) tumor, lymphonodal, or bone metastasis. For subsequent quantitative analysis, the region of interest (ROI) sufficiently large for covering the whole lesion was inserted over each pathological lesion, and the focus on SUVmax and SUVmean of each lesion was calculated by the workstation based on the ROI (15). The SUVmax threshold of 45% was used to obtain agreement with the contour of the lesions on CT, compensating for activity spillover as suggested by Schmuck et al. (16). Since SUV is a measure of uptake of PSMA to tumor foci, it cannot be applied for assessing the overall metabolism of the entire tumor tissue. Consequently, we introduced the volumetric parameters like PSMA-TV (16) and TL-PSMA, referring to the product of PSMA-TV and the SUVmean of the lesion. The concepts of these molecular volumes are derived from FDG imaging, and the calculation of PSMA-TV equals to molecular tumor volume (MTV) (11). At the same time, the calculation of TL-PSMA is equivalent to the total lesion glycolysis (TLG). We know that specific combinations of the radioactive tracer manufacturers, system suppliers, reconstruction techniques, uptake time, post-processing software, and the time between injection of radiotracer and scan will cause bias. Thus, P/M ratio was calculated to eliminate the abovementioned bias by dividing SUV with prostate lesion by SUV with the same cross-sectional level of psoas major muscle.

### Statistical Analysis

Since our sample size is less than 100, we adopted the Shapiro–Wilk test to confirm the normal distribution of data. Normal distribution data were represented by mean ± standard deviation (SD), and parameters with non-normal distribution were represented by median (interquartile range). For classified variables, we used frequency and percentage to describe the data. One-way ANOVA or the Kruskal–Wallis test was conducted for differences between two or more groups, as appropriate. The Mann–Whitney *U* test was adopted to compare between groups when the data were not normally distributed. Otherwise, Student’s *t*-test was adopted for comparison of the difference between two groups. Intraprostatic parameters like MTV, TLG, PSMA-TV, TL-PSMA, and P/M ratio were continuous variables, and Spearman correlation analysis was conducted to study the correlation between intraprostatic parameters and clinical parameters such as PSA levels. Through the area under the curve (AUC) calculation, the performance of the above parameters in distinguishing patients from different risk groups was analyzed. Two-tailed tests were implemented, and *P*-value <0.05 was regarded as statistically significant. SPSS 25.0 statistical software was adopted for all research data analysis.

## Results

### Intraprostatic PSMA Uptake of ^18^F-DCFPyL PET/CT and Clinical Data

**Figure 1:**
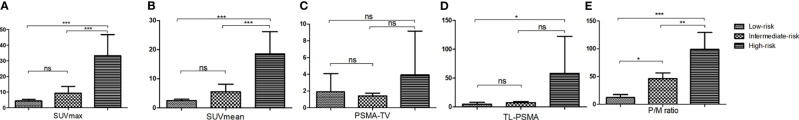
Five common parameters of intraprostatic prostate-specific membrane antigen (PSMA) uptake in newly diagnosed prostate cancer (PCa) patients of different risks. **(A)** Maximal standardized uptake value (SUVmax), **(B)** SUVmean, **(C)** PSMA-TV, **(D)** TL-PSMA, and **(E)** prostate/muscle (P/M) ratio were respectively compared between the low-risk group and intermediate-risk group or high-risk group. Each parameter is presented as mean ± SD. NS means no significance; * means *P <* 0.05; ** means *P <* 0.01; *** means *P <* 0.001.

**Figure 2:**
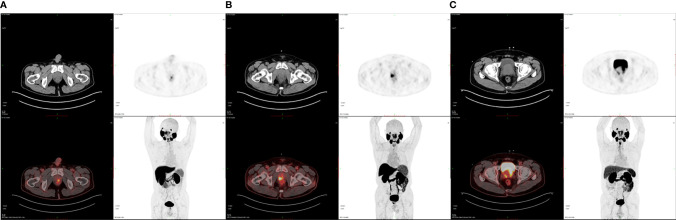
Representative ^18^F-DCFPyL PET/CT images of PCa patients at various risks. **(A)** SUVmax is 4.04 and the P/M ratio is 10.12 in a low-risk PCa patient. **(B)** SUVmax is 10.61 and the P/M ratio is 35.36 in an intermediate-risk PCa patient. **(C)** SUVmax is 30.94 and the P/M ratio is 91.26 in a high-risk PCa patient.

Finally, we enrolled 62 patients newly diagnosed as PCa for ^18^F-DCFPyL PET/CT examination in our institution without previous local or systemic therapy. Among them, 18 patients were at low risk, 12 at intermediate risk, and 32 at high risk of PCa based on the D’Amico scale. The demographic and clinical data of patients are shown in **Table 1**. No statistical significance was observed in PCa patients of different risks in terms of age, BMI, and diabetes as well as hypertension history (*P* > 0.05), demonstrating comparability of these clinical data among the groups. **Table 2** shows the intraprostatic PSMA uptake of ^18^F-DCFPyL PET/CT in different risk groups. Significantly decreased levels of SUVmax, SUVmean, TL-PSMA, and P/M ratio were observed in the low- or intermediate-risk groups or both, compared with the high-risk group. However, only P/M ratio significantly elevated in patients with intermediate risk [mean ± SD (median): 46.58 ± 9.74 (45.27), *P* = 0.042] or high risk [98.95 ± 38.83 (97.52), *P* < 0.001], compared with low-risk patients [12.33 ± 5.93 (9.81)]. Therefore, the P/M ratio has the possibility to become a diagnostic uptake parameter to discriminate among patients with newly diagnosed PCa at low, intermediate, and high risks. **Figure 1** reveals the SUVmax, SUVmean, PSMA-TV, TL-PSMA, and P/M ratio of PCa patients. What stands out in this figure is the significant difference in the P/M ratio between patients at low risk and patients at intermediate risk. **Figure 2** shows the representative images of ^18^F-DCFPyL PET/CT of PCa patients at different risks, and SUVmax and P/M ratio increased obviously with increasing risk. No statistical difference was exhibited among PCa patients at low, intermediate, and high risks in terms of PSMA-TV.

**Table 1:** Baseline characteristics of enrolled patients.

	Low risk	Intermediate risk	High risk
No. of patients	18	12	32
Age (yr), mean ± SD	68 ± 4.34	61 ± 7.79	68.25 ± 6.83
BMI, mean ± SD	24.86 ± 2.68	24.22 ± 3.21	23.89 ± 1.81
History of diabetes, *n* (%)	5 (28)	3 (25)	9 (28)
History of hypertension, *n* (%)	5 (28)	4 (33)	8 (25)
GS, mean ± SD	6.5 ± 0.71	7 ± 0	8.75 ± 0.87
PSA (ng/ml), median (IQR)	9.05 (7.88, 9.25)*	14.65 (7.62, 16.70)*	45.59 (27.17, 69.38)*

No., number; yr, year; SD, standard deviation; BMI, body mass index; GS, Gleason score; PSA, prostate-specific antigen; IQR, interquartile range.

* means P < 0.05.

**Table 2:** ^18^F-labeled PSMA PET/CT parameters in newly diagnosed prostate cancer patients of different risks.

		SUVmax	SUVmean	PSMA-TV	TL-PSMA	P/M ratio
Low risk	Median, mean, SD	4.15, 4.34, 1.06	2.42, 2.46, 0.49	1.04, 1.91, 2.14	3.03, 4.26, 3.87	9.81, 12.33, 5.93
Intermediate risk	Median, mean, SD	8.01, 9.25, 4.45	4.53, 5.46, 2.66	1.39, 1.43, 0.31	6.91, 7.27, 2.07	45.27, 46.58, 9.74
	*P vs*. low risk	0.3	0.291	0.864	0.929	0.042
High risk	Median, mean, SD	32.57, 33.37, 13.43	18.41, 18.51, 7.61	2.66, 3.92, 5.24	40.04, 58.03, 64.36	97.52, 98.95, 38.83
	*P vs*. low risk	<0.001	<0.001	0.345	0.041	<0.001
	*P vs*. intermediate risk	<0.001	<0.001	0.315	0.094	0.001

P-values were calculated by the Mann–Whitney U test or Kruskal–Wallis test when the data do not conform to the normal distribution, and P-values were calculated by one-way ANOVA test or Student’s t-test when the data conform to the normal distribution.

SUVmax, maximal standardized uptake value; SUVmean, mean standardized uptake value; PSMA-TV, PSMA-derived tumor volume; TL-PSMA, total lesion PSMA; P/M ratio, prostate-to-muscle ratio; SD, standard deviation.

### Predictive Value of P/M Ratio in Risk Classification of PCa

**Figure 3:**
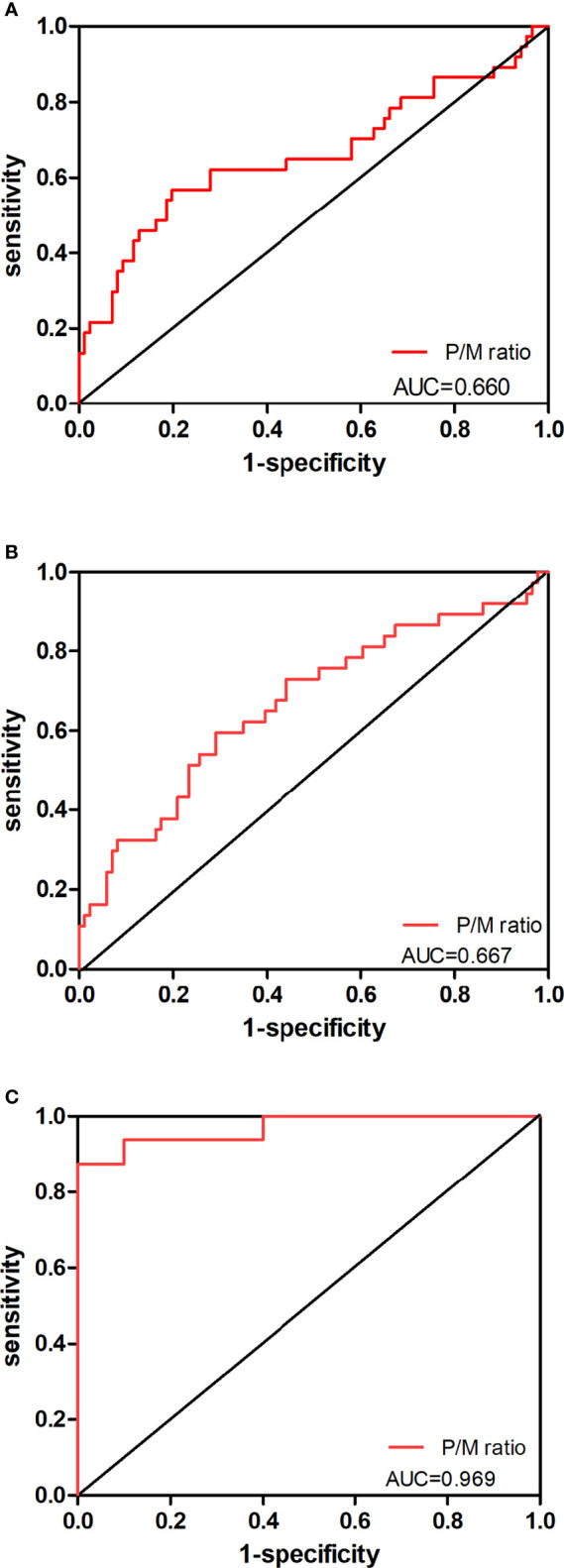
Predictive value of P/M ratio in the risk classification of prostate cancer. The ROC curves for P/M ratio in **(A)** PCa patients at low-risk level versus those at intermediate-risk level, **(B)** PCa patients at intermediate-risk level versus those at high-risk level, and **(C)** PCa patients at low-risk level versus those at high-risk level are shown.

The AUC of receiver operating characteristic (ROC) analysis and the cutoff of the five abovementioned parameters were calculated. A 28.52 threshold for P/M ratio can detect 58.4% of low-risk PCa cases with 76.1% specificity from intermediate-risk patients. At the same time, we used the cutoff for the P/M ratio (>71.43) to discriminate between patient groups at intermediate and high risks with a sensitivity of 57.5% and a specificity of 77.9%. Besides, when the P/M ratio was applied to distinguish patients at low risk from those at high risk, the cutoff of 39.23 was optimal in our study. **Figure 3** reveals the ROC curves for P/M ratio in different risk groups. Interestingly, the P/M ratio revealed the diagnostic sensitivity of 89% and the diagnostic specificity of 100% (ROC AUC = 0.969) for the group at low risk versus the group at high risk.

### Correlation of P/M Ratio and PSA Levels in Patients

**Figure 4:**
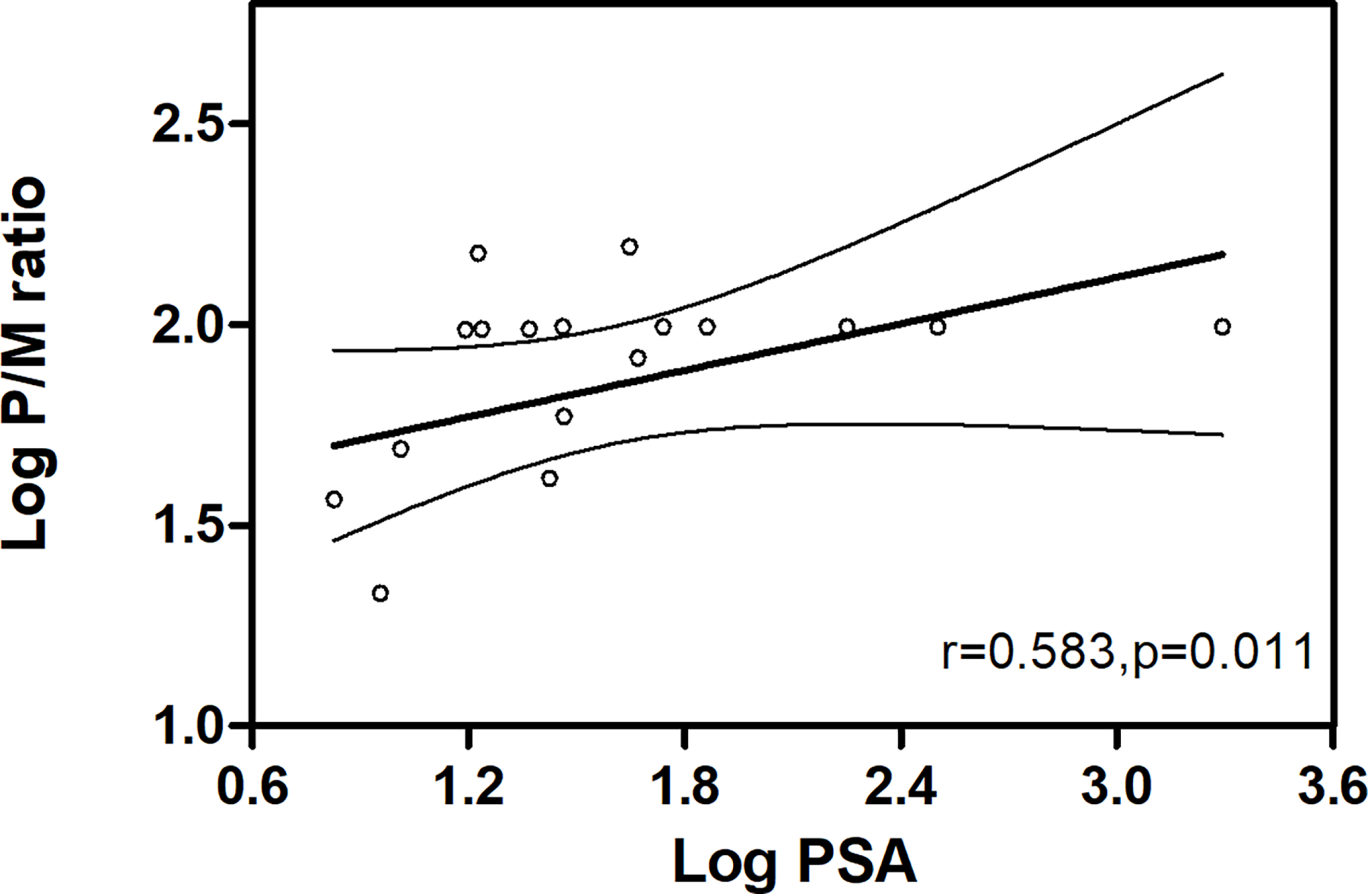
Positive correlation between P/M ratio and PSA levels in PCa patients.

According to our study, a significant difference was exhibited in P/M ratio between any two of the three groups in **Table 2** (*P* < 0.05 for all). Thus, we analyzed the correlation of P/M ratio with PSA among PCa patients. As shown in **Figure 4**, P/M ratio exhibited a positive association with PSA in all risk patients (*r* = 0.583, *P* = 0.011) in accordance with Spearman correlation analysis.

## Discussion

Although the treatment of PCa has been progressing for the past few years, the long-term survival rate of the patients was still determined by the accurate staging of PCa. Hence, new strategies for diagnosing and classifying patients with PCa are urgently needed (6). As the ideal PCa imaging method, PSMA PET/CT has attracted more and more attention recently because of its high sensitivity and specificity in PCa diagnosis (17). Previously, PSMA PET/CT had been extensively applied in the qualitative diagnosis of PCa patients, especially in identifying the sites of disease recurrence with increased PSA after the first treatment (18). Few researchers focused on the effect of PSMA PET/CT quantitative parameters on risk classification of newly diagnosed patients without treatment (19).

In this study, we compared five quantitative metabolic parameters of PSMA PET/CT, namely, SUVmax, SUVmean, PSMA-TV, TL-PSMA, and P/M ratio, in newly diagnosed PCa patients of different risks according to the D’Amico criteria. Among the five parameters mentioned above, SUVmax, SUVmean, and P/M ratio were significantly decreased in patients at low or intermediate risk compared with those at high risk, which had a similar result as in a previous study (19). However, in our research and the study of Koerber et al. (19), we find a significant overlap in SUVmax between the groups at low risk and at intermediate risk. As a result, there will be a high false-positive rate when SUVmax is used as a grouping index. Here, our results suggest that P/M ratio is a reliable parameter of intraprostatic PSMA uptake to distinguish patients at low risk and intermediate risk (*P* = 0.042), which was seldom reported before.

In addition, the positive correlation between P/M ratio and PSA values in all PCa patients further supports the hidden value of P/M ratio as a selective grouping index for PCa (**Figure 4**). PSMA is a physiologically expressed protein in different tissues affected by race, region, injection dose of tracer, and high degree heterogeneity of prostate cancer (20), as indicated by measuring a wide range of SUVmax of cancer. In comparison, P/M ratio can reduce the interference of the above factors to a certain extent and magnify the differences between different patients (**Figure 1**). According to the results of univariate analyses, P/M ratio is a more promising parameter for risk classification of prostate cancer than SUVmax, and the performance of the P/M ratio in distinguishing patients from different risk groups with *c*-statistics showed values of 0.660, 0.667, and 0.969, respectively. Multiple small lesions are the pathological features of prostate carcinoma, and some lesions of PCa are even less than 5 mm in size in low- and intermediate-risk patients (21). Given TL-PSMA = SUVmean * PSMA-TV, the diagnostic capabilities of TL-PSMA and PSMA-TV are decreased due to the influence of volume of lesions (12).

Recently, PSMA PET/CT has received more and more attention and extensive discussion. PSMA PET/CT has a high accuracy in diagnosing PCa even at low PSA levels (22); therefore, the rate of false-negative findings in patients with newly diagnosed PCa is expected to be low. However, due to the constraints of cost, equipment, and production capacity, it is not easy to popularize this imaging modality at present. Our study enriches the related research and provides some basis for the follow-up PSMA PET/CT research. Meanwhile, to our knowledge, few studies have mentioned and proved the diagnostic efficacy of P/M ratio in risk classification of exclusively untreated, newly diagnosed prostate cancer. Finally, due to the deficiency of relevant prospective clinical research data, larger sample-sized and well-designed studies are further required to verify our results.

## Conclusion

The five commonly used parameters of intraprostatic PSMA uptake were compared in different PCa risk groups, and SUVmax, SUVmean, and P/M ratio might assist in distinguishing patients at low or intermediate risk from those at high risk. However, when comparing patients at low risk and those at intermediate risk, only the P/M ratio is statistically significant between groups. According to our data, P/M ratio appears to be a potential grouping index like Gleason score and PSA in the risk classification of PCa which was further proved by its positive correlation with PSA levels.

## Data Availability Statement

The original contributions presented in the study are included in the article/supplementary material. Further inquiries can be directed to the corresponding author.

## Ethics Statement

The studies involving human participants were reviewed and approved by the Ethics Committee of Xinhua Hospital Affiliated to Shanghai Jiao Tong University School of Medicine. The patients/participants provided their written informed consent to participate in this study. Written informed consent was obtained from the individual(s) for the publication of any potentially identifiable images or data included in this article.

## Author Contributions

QC and JK participated in the study design, case enrollment, PET/CT acquisition, image processing, clinical management, statistical analysis, manuscript writing, and submission. SZ, TL, and ZZ participated in PET/CT acquisition, image processing, statistical analysis, and manuscript writing. HF participated in the study review, image processing, and case enrollment. LZ and SQ participated in case enrollment. All authors contributed to the article and approved the submitted version.

## Conflict of Interest

The authors declare that the research was conducted in the absence of any commercial or financial relationships that could be construed as a potential conflict of interest.

## Publisher’s Note

All claims expressed in this article are solely those of the authors and do not necessarily represent those of their affiliated organizations, or those of the publisher, the editors and the reviewers. Any product that may be evaluated in this article, or claim that may be made by its manufacturer, is not guaranteed or endorsed by the publisher.
